# Health Threats of All Stripes

**DOI:** 10.3201/eid1807.AC1807

**Published:** 2012-07

**Authors:** Polyxeni Potter

**Affiliations:** Centers for Disease Control and Prevention, Atlanta, Georgia, USA

**Keywords:** art science connection, emerging infectious diseases, art and medicine, Gene Davis, Niagara Knife, Health threats of all stripes, post painterly abstraction, Washington Color Group, International Health Regulations, about the cover

**Figure:**
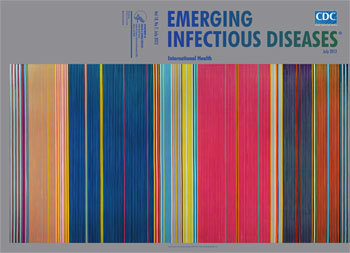
**Gene Davis (1920–1985) *Niagara Knife* (1967) Acrylic on canvas (294.6 cm × 546.1 cm)** High Museum of Art, Atlanta, Georgia, USA. Gift of Turner Broadcasting System, Inc.

“I just decided to do a stripe painting, just to be outrageous,” Gene Davis said, pondering the origins of his iconic works. “Let’s see if I can’t do something that goes in the opposite direction from painterly abstraction.” This decision “to get away from painterliness” and “move somewhere else” was at the heart of his art. “It’s something, you know, that shakes them up. It’s not a painting of a bouquet of flowers.”

“I’ve never been a realist artist…. I haven’t gone through the usual classical training at all. I just bypassed the entire issue. And I’m not sorry.” Davis admitted reluctance to being bossed or instructed and professed being a free operator. All the same and despite the absence of academic training, he came to art early in life. “When I was 8, 9 years old, somewhere in that vicinity, I used to do little childlike drawings and send them in to the Washington Post ‘children’s page’… and they thought enough of them to publish them…. And then I took… a drawing course in high school.” Later in his career he taught art at the Corcoran School of Art and Design and for a time at American University, Skidmore College, and the University of Virginia.

A native Washingtonian, Davis frequented art venues, particularly the Phillips Collection. “The small masterpieces of Paul Klee… made an unforgettable impression on me, and I can remember being equally smitten with the complex color harmonies of Bonnard.” But his interest did not peak until his late 20s, “Because during my early 20s, I was a very happy newspaper man. I covered the White House for 5 years.” This career included stints as sports writer for the now defunct Washington Daily News and work for United Press International and the New York Times―as a copy boy “a real elitist job to have, because it was a stepping stone to the reportorial.” “I earned my living as a writer for something like 35 years before I really was successful enough as an artist to quit my job and to paint full time. And that took place in 1968.”

Davis did not “jump into the art stream” until 1949. “I started after having read an article in the New York Times about van Gogh that turned me on.” He did not join the local art scene until 1950 when he met noted Washington artist and curator Jacob Kainen, who became his mentor and introduced him to Morris Louis and Kenneth Nolan…. “In those days, the big issue was whether you were going to be a realist or an abstractionist…. I leaped right in as an abstractionist.” “What really impressed me about the abstract expressionists [Jackson Pollock and his circle] was the degree to which you could deemphasize skill and still say something that had tremendous intensity…. It’s the ‘what’ of it more than the ‘how’ of it.”

But soon “All the art departments―college art departments―were grinding out little de Koonings and Pollocks…. So in that climate, it seemed that… there was no place to go.” Young painters, Davis among them, were looking for change. “Frank Stella, Noland, myself. There’s a whole group of them ―Ellsworth Kelly.” Their new direction was soon labeled “post-painterly abstraction,” and a group of artists who had not intended to band together began to be referred to as the Washington Color School, their bold work anticipating later movements. “I’d be the last person in the world to claim that Washington’s art influenced Pop Art but I think things were in the air. And they had bright, brazen colors just like we did. There was something, a common denominator that went through the ‘60s. It was an exciting period. The Kennedy era, optimism was in the air, excitement, campus rebellion―all that stuff was all―you can’t isolate any of it.”

“What you see is what you see,” proclaimed Frank Stella, expressing the period’s preoccupation with art concerned only with the direct experience of color and form. Davis also elaborated on the content of color and form. “I have very, very strong subject matter in my work, which is stripes.” Invested with enough intensity, “A stripe is just as real as a… flower…. There’s no such thing as a painting about nothing…. For example, if you look at 17-th century Dutch art, you’ll see that there are endless numbers of painters who painted the same subject matter as Rembrandt―these middle class people with their big hats and their long collars and all that. So, Rembrandt’s great, and most of those people are eminently forgettable. What makes the difference? It isn’t the subject matter, obviously. It’s the form.”

*Niagara Knife,* on this month’s cover, is one of Davis’ stripe paintings, a hallmark of his work for 20 years―the stripe as form. He painted mostly vertical stripes, because horizontal ones “carry the illusion of landscape.” He painted color stripes on the street leading to the Philadelphia Museum of Art and a parking lot in Lewiston, New York, turning them into massive works of art. He never planned more than a few stripes ahead. He improvised, allowing each color to inspire the next.

“I play by eye in the same way that a jazz musician plays by ear.” In addition to form, color was of great interest to Davis. Color and interval―the distance between things, as in music. “Music is an art of sound interval, time interval, and painting―my painting―is an art of space intervals. One is time, one is space.” A frustrated musician, he often referred to music as a way of discussing his work. “If you have a painting which has all half-inch stripes in it, multi-color, and you put a bright red over here, and another bright red over there, no more of those bright reds in the entire painting, there’s an interval established between the two reds. Because all the other colors in the painting will be something else. But these two relate.”

“I paint to surprise myself.” Davis believed that shocking or even offending the viewer had an energizing effect. “Ambiguity interests me.” This could be created by the contrast of opposites. “It’s a little like Mozart, who was a master of ambiguity in that his works can often be regarded as little tinkling, felicitous things, but there’s a strong note of melancholy running throughout. You get that melancholy plus felicity and it creates ambiguity.”

The breadth of a line, the distance between colors, and the interaction of colors create an optic and kinetic effect and an architectural complexity in Davis’ work that appear analytical, mathematical. Yet, it is all “intuitive and romantic.” “I’m a real shoot-from-the-hip artist.” The work invites personal interpretation, teasing the eye and challenging it to grasp the total, vibration and all.

In that each stripe is individually executed to be viewed at once alone and in conjunction with the others, Davis’ *Niagara Knife* is not unlike the effort addressed in this month’s issue of Emerging Infectious Diseases: public health at the global level. Each laboriously painted thin or thick stripe, each narrow or wide interval, each lyrical color combination a nation; marching bands of color a dazzling array of diversity and separateness; and altogether as Davis intended them, a bright ensemble, a symphony of color, a public health collaboration as spectacular as any bouquet of flowers.

“Painting stripe paintings is a vigorous kind of thing. I’ve got at least to be down on my hands and knees,” Davis explained to those curious about his craft. Laboriously, line by line, the painting becomes an integrated total. The same rigor certainly applies to drafting any international regulations, including those intended to protect public health. Outbreak by outbreak, experience with Public Health Emergencies of International Concern delineates what requires international reporting to improve global health and emergency response. Because, while individual stripes are drawn on the canvas of global health as nations agree to report to the World Health Organization those health events of concern to international health, the total picture is harder to assemble. Distinguishing which health events pose international health threats is at times as ambiguous as a Davis painting, and therefore, implementation of the International Health Regulations has not yet realized its full potential.

While ambiguity can energize a work of art, it can upset a regulatory document. Generally avoided for its capacity to introduce uncertainty, ambiguity represents the human factor, addressed in the regulations by a decision instrument to guide subjective judgment. Improving the validity of the instrument, along with clarifying measurable goals and progress indicators, promises to overcome some of the ambiguity, pulling individual stripes into bands and the bands into a full form the colors of international health.
